# Effect of polyhexamethylene biguanide-coated central venous catheters on bacterial colonization in cancer patients undergoing abdominal surgery: a randomized controlled trial

**DOI:** 10.3389/fmed.2025.1507352

**Published:** 2025-02-19

**Authors:** Jun Dong, Yong Yang, Qi Li, Jia-Xuan Xu, Yan-Fen Shen, Hong-Zhi Wang

**Affiliations:** ^1^Key Laboratory of Carcinogenesis and Translational Research (Ministry of Education/Beijing), Intensive Care Unit, Peking University Cancer Hospital & Institute, Beijing, China; ^2^Key Laboratory of Carcinogenesis and Translational Research (Ministry of Education/Beijing), Department of Clinical Laboratory, Peking University Cancer Hospital & Institute, Beijing, China

**Keywords:** central venous catheter (CVC), catheter-related bloodstream infection (CRBSI), bacterial colonization, PHMB coating, cancer patients, abdominal surgery

## Abstract

**Background:**

Central venous catheters (CVCs) are widely used in critically ill patients, including cancer patients, but are associated with complications such as catheter-related bloodstream infections (CRBSIs). This study evaluates the effectiveness of polyhexamethylene biguanide (PHMB)-coated CVCs in reducing catheter-tip bacterial colonization in cancer patients undergoing abdominal surgery.

**Methods:**

A prospective, randomized, monocentric clinical trial was conducted at Peking University Cancer Hospital from March 2017 to April 2019. Surgical cancer patients requiring CVCs were randomized into two groups: a PHMB-coated CVC group (Certofix^®^ protect) and a standard CVC group (Certofix^®^). The primary outcome was catheter tip bacterial colonization, and the secondary outcomes included catheter retention time and hospital length of stay.

**Results:**

A total of 1,185 patients were included in the analysis. The incidence of catheter tip bacterial colonization was 2.5% in the PHMB-coated group and 4.2% in the standard CVC group (*p* = 0.10). Hospital length of stay was significantly shorter in the PHMB-coated group (*p* < 0.001). Subgroup analysis showed reduced bacterial colonization in male patients in the PHMB-coated group (*p* = 0.04).

**Conclusion:**

Polyhexamethylene biguanide-coated CVCs did not significantly reduce catheter tip bacterial colonization in the overall population but showed a beneficial effect in male cancer patients undergoing abdominal surgery. In clinical practice, it is necessary to consider various factors when selecting the type of catheter.

**Clinical trial registration:**

No. chiCTR-IPR-16010027.

## 1 Introduction

Central venous catheters (CVCs) are essential for hemodynamic monitoring, medication administration, and parenteral nutrition in critically ill patients. However, they are associated with complications such as mechanical injury, infection, and thrombosis, which increase hospital costs, prolong hospital stays, and elevate mortality rates (1–3). Catheter-related bloodstream infection (CRBSI) is one of the most common and serious complications, leading to increased mortality and hospital costs (4). Cooper and Hopkins (5) demonstrated that bacterial colonization on CVCs could occur as early as 1 h after insertion, and these colonizing organisms are closely associated with the development of CRBSI. Clinical consequences showed that the probability of developing catheter colonization was approximately 24.7% in patients given standard CVCs in place for approximately 5 to 11 days (3). Recent studies have also indicated that among patients with central venous catheters, 48.6% have microbial colonization at the tip of the catheter. The reason for microbial colonization is thought to be related to the formation of a biofilm within the catheter lumen, which facilitates microbial attachment (6, 7). However, catheter microbial colonization is not a necessary and sufficient condition for CRBSI. The occurrence of CRBSI is also associated with factors such as host immune suppression and improper catheter management (8). The majority of studies indicate that more than half of CRBSI cases are caused by staphylococci, followed by Gram-negative bacilli and Candida. Among the CRBSI cases caused by Gram-negative bacilli, approximately 30% of cases are attributable to these pathogens, with 50% exhibiting multidrug resistance (6, 9). Recommendations by the Centers for Disease Control and Prevention (CDC) to prevent CRBSI include education and training, maximal sterile barrier precautions, skin preparation, catheter site dressing regimens, and the use of antimicrobial-impregnated catheters (1). Antibacterial coatings such as those containing polyhexamethylene biguanide (PHMB) have been proposed as an effective method to prevent bacterial colonization and subsequent CRBSI in both *in vitro* and animal models (10). This study aimed to assess the effectiveness of PHMB-coated CVCs in reducing catheter tip bacterial colonization in cancer patients undergoing abdominal surgery.

## 2 Methods

### 2.1 Study design

This prospective, randomized, monocentric clinical trial was conducted at Peking University Cancer Hospital and Institute in China from March 2017 to April 2019. Cancer patients who underwent abdominal surgery and required a CVC were randomly assigned to either the PHMB-coated CVC group or the standard CVC group. The study was approved by the Institutional Review Board of Peking University Cancer Hospital (NO. 2016KT28) and registered at the Chinese Clinical Trial Registry (No. chiCTR-IPR-16010027). Written informed consent was obtained from all patients. This study was funded by the B. Braun Anesthesia Science Research Foundation (BBFD-2015-16).

### 2.2 Participants

**Figure 1:**
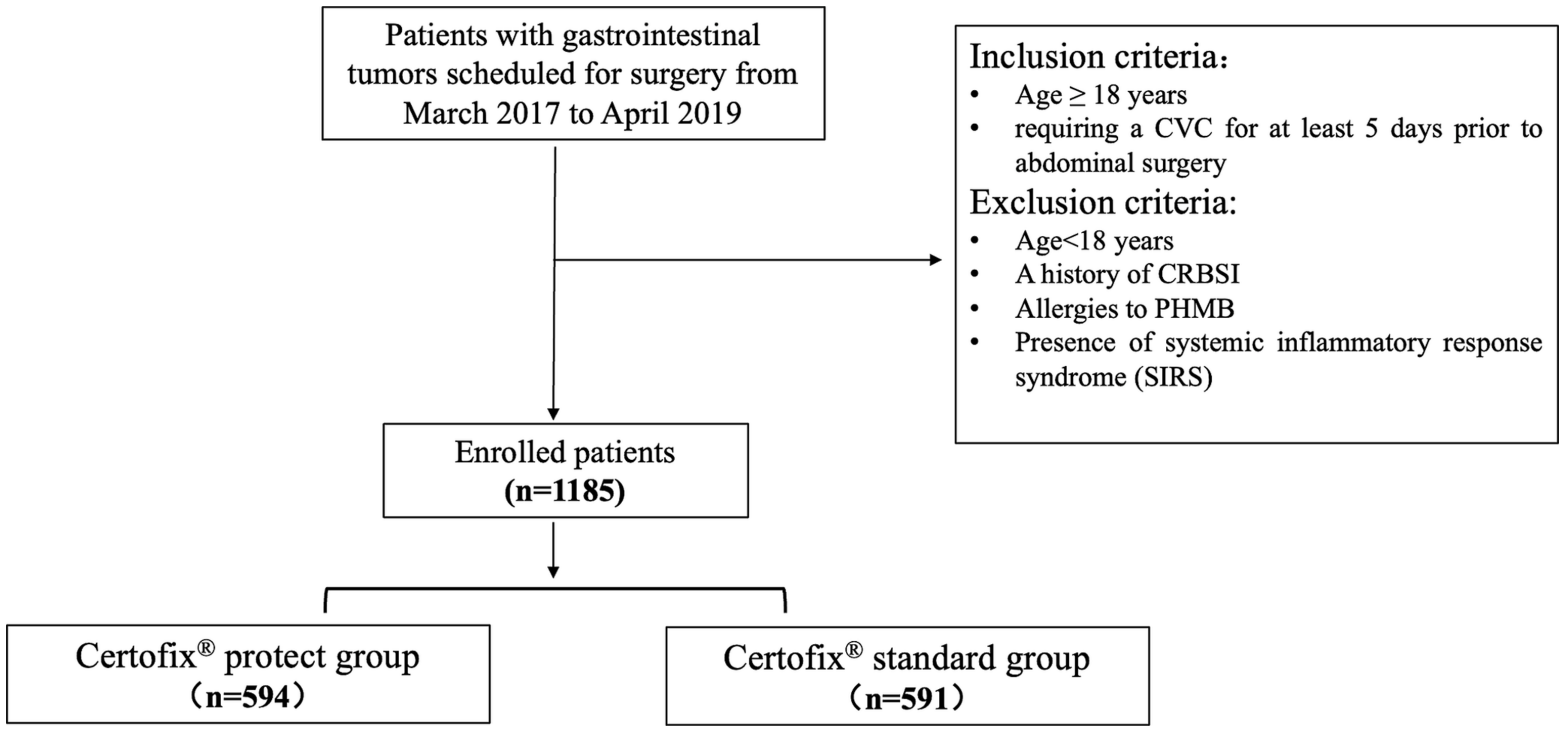
Grouping flowchart.

Eligible participants included adult patients (≥18 years) requiring a CVC for at least 5 days before abdominal surgery. The exclusion criteria included ① patients < 18 years; ② a history of CRBSI; ③ allergies to PHMB; and ④ the presence of systemic inflammatory response syndrome (SIRS) (Figure 1).

A sample size of 442 patients was calculated, assuming a 20% reduction in bacterial colonization. A total of 1,185 patients were included, with 594 in the PHMB-coated group and 591 in the standard CVC group. Therefore, the inclusion of more than 590 patients in each group met the minimum sample size requirement.

### 2.3 Interventions

Eligible patients were randomly divided, using a random number table, into two groups: Patients in the PHMB-coated group received Certofix^®^ protect CVCs (Certofix^®^ protect, B. Braun, Germany) coated with PHMB, and the standard CVC group received Certofix^®^ CVCs without PHMB (Certofix^®^, B. Braun. Germany). All catheter insertions were performed by trained ICU doctors and nurses at the vascular access center adhering to standard infection control measures (1). Strict adherence to the regulations was maintained throughout the procedure, and the skin was disinfected using 2% chlorhexidine and 75% alcohol for 30 s. The catheter was secured to the skin with sterile sutures to prevent any bending and then covered with a 3M Tegaderm-1679 clear dressing. For catheter maintenance, including disinfection with 2% chlorhexidine and 75% alcohol, dressing changes, and catheter flushing, these procedures were performed every 5–7 days. Both patient groups were treated by the same team of highly trained doctors and nurses, ensuring consistent disinfection and treatment methods. In accordance with departmental protocols, the doctor’s attempts at puncture were limited to a maximum of three. If unsuccessful after three attempts, the puncture site was changed under ultrasound guidance. This approach, utilizing ultrasound-guided central venous catheterization (CVC), has been shown to significantly improve puncture success rates and reduce complications such as infection and pneumothorax (11).

Previous studies have demonstrated that CRBSI is associated with the site of catheter implantation. Specifically, the infection rate for central venous catheters (CVC) inserted into the subclavian vein is lower than that for those implanted in the internal jugular vein (12). In both groups, the catheters were inserted into the subclavian vein. After catheterization, regional treatment with sterile gauze was performed every 5–7 days. When the catheter was no longer needed, it was removed and a 5-cm distal catheter segment was sent to the laboratory for semi-quantitative culture using the roll plate method. The collected specimens from the distal catheter segment were inoculated and cultured on bacterial screening plates, and bacterial identification and antimicrobial susceptibility testing were performed using the VITEK2 automated bacterial identification and susceptibility testing system (both the plates and the instrument were provided by bioMérieux, France). In cases where patients developed a fever and catheter-related bloodstream infection (CRBSI) could not be ruled out, the catheter was removed. Before catheter removal, blood samples from both the catheter and a peripheral vein, along with the 5-cm distal catheter segment, were sent for laboratory culture. In addition, if a surgical infection was suspected after the operation, the physician conducted an etiological examination of the patient’s abdominal drainage fluid.

CRBSI is defined as the isolation of the same organism from both a semi-quantitative or quantitative culture of a catheter segment and separate percutaneous blood cultures, with no other identifiable source of infection (1). Gram stain and culture of the exudate should be performed if inflammation or exudation occurs at the puncture site. The criteria for catheter removal included (1) the decision of the assessing doctors that the catheter was no longer necessary and (2) the inability to exclude CRBSI in patients presenting with fever or other sepsis symptoms.

### 2.4 Outcomes

The primary outcome was the incidence of catheter tip bacterial colonization, defined as ≥15 CFU in a semi-quantitative culture, as per Maki’s criteria (13).

The secondary outcomes included catheter retention time, hospital length of stay, and the incidence of CRBSI, defined as the isolation of the same organism from a catheter segment and separate blood cultures, with no other identifiable source of infection.

### 2.5 Statistical analysis

Data were analyzed using IBM SPSS Statistics, version 20. Categorical variables are presented as frequencies and percentages, and comparisons were made using the chi-square test. Continuous variables are expressed as mean ± standard deviation and were compared using the Student’s *t*-test. A *p*-value of less than 0.05 was considered statistically significant.

## 3 Results

A total of 1,185 cancer patients undergoing abdominal surgery were included in the final data analysis: 594 patients in the Certofix^®^ protect group and 591 patients in the standard Certofix^®^ group. Of these, 61.10% (724/1185) of the patients were diagnosed with gastric cancer and 38.90% (472/1185) were diagnosed with intestinal cancer. The overall catheter tip bacterial colonization rate for all patients was 3.38% (40/1185) (colonization bacteria from the 40 cases in Table 1), while the Certofix^®^ protect group showed a rate of 2.5% and the standard Certofix^®^ group showed a rate of 4.2%. No significant difference was observed between the two groups (*p* = 0.10 Table 2). Male patients had a 4.23% catheter-tip bacterial colonization rate (34/803), while female patients had a 1.57% (6/382) catheter-tip bacterial colonization rate. However, patients in the Certofix^®^ Protect group had a shorter hospital length of stay, approximately 14 days, than those in the standard Certofix^®^ group (*p* < 0.001; Table 2).

**Table 1:** Colonization bacteria from the 40 cases.

Colonization bacteria (*n* = 40)	*n* (%)
Coagulase-negative staphylococci	27(67.5)
*Staphylococcus epidermidis*	19
Human *Staphylococcus*	3
Hemolytic *Staphylococcus*	3
*Staphylococcus capitis*	2
*Staphylococcus aureus*	2(5)
*Streptococcus constellatus*	4(10)
*Escherichia coli*	3(7.5)
*Klebsiella pneumoniae*	3(7.5)
*Acinetobacter baumannii*	1(2.5)

**Table 2:** Bacterial colonization in all enrolled patients.

	Certofix^®^ Protect (*n* = 594)	Certofix^®^ *n* = 591)	*P*-value
Age (years)	59.96 ± 10.77	59.09 ± 11.10	0.17
Male (*n*, %)	425 (71.5)	378 (64.0)	0.14
Gastric cancer (*n*, %)	365 (61.5)	359 (60.7)	0.20
Bacterial colonization (*n*, %)	15 (2.5)	25 (4.2)	0.10
Catheter retention time (days)	13.12 ± 9.77	12.52 ± 7.31	0.24
95% CI	(12.33,13.91)	(11.93,13.11)	
Length of stay in hospital (days)	13.95 ± 7.92	15.88 ± 10.14	<0.001
95% CI	(13.31,14.58)	(15.06,16.70)	

Analysis by gender revealed that in male patients, the incidence of catheter tip bacterial colonization was lower in the Certofix^®^ protect group than in the standard Certofix^®^ group (2.8% vs. 5.8%, *p* = 0.04; Table 3). However, no significant differences were observed in catheter retention times or hospital length of stay between the two groups in male patients. In female patients, no differences were found in catheter tip bacterial colonization between the two groups, but the hospital length of stay was significantly shorter in the Certofix^®^ protect group than in the standard Certofix^®^ group (*p* < 0.001; Table 3).

**Table 3:** Bacterial colonization in enrolled male patients and female patients.

Patients	Certofix^®^ protect		Certofix^®^	*P*-value
Male patients (*n*, %)	425 (71.5)		378 (64.0)	0.15
Age (years)		60.63 ± 9.85	60.42 ± 10.26	0.77
Bacterial colonization (*n*, %)		2.8 (12)	5.8 (22)	0.04
Catheter retention time (days)		13.16 ± 9.06	12.91 ± 7.613	0.67
95% CI		(12.30, 14.03)	(12.14, 13.68)	
Length of stay in hospital (days)		14.97 ± 8.79	15.92 ± 8.71	0.12
95% CI		(14.13, 15.81)	(15.04, 16.80)	
Female patients (*n*, %)	169 (28.5)		213 (36.0)	0.16
Age (years)		58.28 ± 12.67	56.74 ± 12.11	0.23
Bacterial colonization (*n*, %)		1.8(3)	1.4(3)	1.00
Catheter retention time (days)		13.01 ± 11.398	11.84 ± 6.692	0.21
95% CI		(11.27, 14.74)	(10.94, 12.74)	
Length of stay in hospital (days)		11.37 ± 4.14	15.82 ± 12.30	<0.001
95% CI		(10.74, 12.00)	(14.16, 17.48)	

Among the 1,158 patients in the study, six patients in the standard Certofix® group had their catheters removed early due to suspected CRBSI. Before catheter removal, blood samples from both the catheter and a peripheral vein, along with a 5-cm distal catheter segment and an abdominal drainage sample, were sent for laboratory culture. However, none of the six patients were finally diagnosed with CRBSI (Table 4).

**Table 4:** Six cases removing catheter early.

Case	Blood culture		Catheter-tip culture	Abdominal drainage culture	Survival in hospital	Survival within 90 days
	From catheter	From peripheral vein				
1	*Escherichia coli*	*Escherichia coli*	N	*Escherichia coli*	Y	Y
2	N	N	N	*Escherichia coli*	Y	Missing
3	N	N	*Acinetobacter baumannii*	N	Y	Y
4	N	N	N	N	Y	Y
5	N	*Streptococcus constellatus*	N	*Enterobacter cloacae* *Streptococcus mitis*	Y	N
6	N	N	N	*Enterococcus faecium*	Y	Y

N, negative; Y, yes.

## 4 Discussion

Catheter-related bloodstream infections (CRBSIs) remain a leading cause of morbidity and mortality in critically ill patients, particularly those with cancer. CRBSIs have multiple potential sources, but the most common is thought to be the migration of microorganisms from the patient’s skin along the outer surface of the catheter to the insertion site (14). CRBSI is a serious complication of central venous catheters, often leading to prolonged hospital stays, increased healthcare costs, and higher mortality rates. The majority of CRBSI cases are caused by bacterial infections, with coagulase-negative staphylococci (CoNS) being the most frequently isolated pathogens in clinical studies (8). However, *Staphylococcus aureus*, *Enterococcus* spp., and Gram-negative pathogens such as *Escherichia coli* and *Pseudomonas aeruginosa* are also commonly implicated. In immunocompromised patients, such as those with cancer, *Candida* spp. have emerged as a leading cause of fungal CRBSI (15, 16). Studies have demonstrated that bacterial colonization of the catheter tip is closely associated with the development of CRBSI (5). To reduce the incidence of CRBSI, several preventive strategies have been proposed. The CDC bundle protective strategy for CRBSI includes measures such as hand hygiene and aseptic techniques, thorough skin disinfection with chlorhexidine, using appropriate catheters, selecting suitable catheter insertion sites, employing maximal sterile barriers during catheter insertion, and ensuring the removal of the catheter when it is no longer necessary (17, 18). Among these, catheter coatings, particularly PHMB coating, have gained widespread recognition. PHMB is a cationic polymer that can bind to negatively charged membrane lipids, including those found in Gram-negative and Gram-positive bacteria, thereby increasing membrane permeability (19). As a broad-spectrum antimicrobial, PHMB has been shown to reduce biofilms in wounds and promote wound healing by decreasing wound size (20). More importantly, PHMB has also been demonstrated to inhibit the growth of *Staphylococcus aureus*, a pervasive and significant pathogen in CRBSI (21).

The results of this study are consistent with previous research (22). In approximately 67.5% of cases (27/40), bacterial cultures from catheter tip segments identified CoNS, which are part of the normal skin flora. While these organisms are generally of low virulence, they are increasingly recognized as the most common cause of nosocomial bloodstream infections. Previous studies have shown that in non-neutropenic patients without local symptoms, CRBSI caused by CoNS is often treated with extubation therapy alone, without leading to short-term complications or long-term recurrence (23).

Data from Wisplinghoff et al. (24) indicated that the most common organisms responsible for bloodstream infections (BSIs) were CoNS. They also found that among all potential factors predisposing patients to CRBSI, intravascular devices were the most frequently implicated. In this study, PHMB was shown to reduce catheter tip bacterial colonization in male patients. The higher rate of bacterial colonization in male patients can be attributed to several factors:

1. Studies suggest that male hormones, such as testosterone, can influence the skin’s microbiome. Testosterone increases sebum production, which may create a favorable environment for bacterial growth, particularly for species such as *Staphylococcus aureus* and *Staphylococcus epidermidis* (8).
2. Men generally have more body hair, especially in areas where catheters are placed. This hair can harbor bacteria and impair the effectiveness of disinfection and cleaning procedures, thereby contributing to bacterial colonization (8).
3. Hygiene habits may also play a role. Some studies indicate that men may be less consistent in following hygiene protocols, such as handwashing and catheter site care, which increases the risk of bacterial colonization around the catheter (25).

These above factors collectively contribute to the increased bacterial colonization and infection rates observed in male patients with catheters.

In this study, no significant differences in catheter retention time or hospital length of stay were identified between the two groups, although in female patients, the hospital length of stay in the Certofix^®^ protect group was shorter than that in the standard Certofix^®^ group. Given that many factors can influence hospital length of stay, it is not possible to attribute this difference to PHMB without further targeted studies.

It is noteworthy that the six patients who had their catheters removed early due to suspected CRBSI were all in the standard Certofix^®^ group. Only one of these patients tested positive for catheter tip bacteria colonization, but blood cultures from both the catheter and a peripheral vein were negative. After evaluating clinical symptoms, laboratory tests, including imaging differences, and positive blood cultures, clinicians ultimately determined that the cause of the fever in these patients was abdominal infection rather than CRBSI.

Differential time to positivity (DTP) is one method for the diagnosis of CRBSI, which is also a laboratory diagnostic standard for CRBSI. According to this method, CRBSI is suspected if, after simultaneous blood culture collection from both the central vein and a peripheral vein, the blood culture from the central vein shows growth at least 2 h earlier than that from the peripheral vein, and under the premise of excluding other infections, this bloodstream infection is considered to be CRBSI (26). Obtaining paired catheter and peripheral blood cultures for DTP when the infectious source is unclear may prevent unnecessary line removal and diagnostic tests (27). We ruled out the diagnosis of CRBSI for the six suspicious patients through this method.

### 4.1 Limitations

Our study has several limitations. First, due to the specific patient population in our department, the study focused on cancer patients with a history of abdominal surgery. As such, the findings are not generalizable to all patients undergoing clinical surgery. We also did not fully account for all factors that may affect bacterial colonization, which will be a focus of future research. In our next study, we plan to conduct a comprehensive, in-depth analysis of the factors influencing bacterial colonization after central venous catheter (CVC) insertion and explore potential preventive measures. In addition, this study was conducted at a single center. Expanding the research to multiple centers and including a broader range of cancer patients may yield different results. Finally, we used a semi-quantitative rolling plate test for bacterial colonization confirmation. If the bacterial colonization period is extended, using more precise methods, such as ultrasonic oscillation for quantitative detection, could enhance accuracy and help to identify potential hidden risks of catheter-related infections.

## 5 Conclusion

Bacterial colonization of CVC after abdominal surgery in cancer patients is affected by multiple factors. PHMB-coated CVCs did not significantly reduce catheter tip bacterial colonization in cancer patients undergoing abdominal surgery. However, they may reduce bacterial colonization in male patients, and further studies with larger sample sizes are required to explore these findings. This result also indicates that PHMB-coated catheters have limited advantages in preventing catheter-related infections. Therefore, clinicians should consider factors such as cost, durability, and patient-specific characteristics when selecting a catheter. Furthermore, strict adherence to sterile procedures and enhanced catheter maintenance are necessary to reduce the risk of catheter-related infections.

## Data Availability

The data of this study can be obtained from the corresponding author upon reasonable request.
